# First Report of the Occurrence of *Trichinella*-Specific Antibodies in Domestic Pigs in Central and Eastern Uganda

**DOI:** 10.1371/journal.pone.0166258

**Published:** 2016-11-21

**Authors:** Kristina Roesel, Karsten Nöckler, Maximilian P. O. Baumann, Reinhard Fries, Michel M. Dione, Peter-Henning Clausen, Delia Grace

**Affiliations:** 1 Institute for Parasitology and Tropical Veterinary Medicine, Department of Veterinary Medicine, Freie Universität Berlin, Berlin, Germany; 2 Food safety and zoonoses program, International Livestock Research Institute, Nairobi, Kenya; 3 Department Biological Safety, Federal Institute for Risk Assessment, Berlin, Germany; 4 FAO Reference Centre for Veterinary Public Health, Department of Veterinary Medicine, Freie Universität Berlin, Berlin, Germany; 5 Institute for Meat Hygiene, Department of Veterinary Medicine, Freie Universität Berlin, Berlin, Germany; 6 Animal science for sustainable productivity program, International Livestock Research Institute, Kampala, Uganda; Instituto de Diagnostico y Referencia Epidemiologicos, MEXICO

## Abstract

Previous research on trichinellosis in Africa focused on isolating *Trichinella* from wildlife while the role of domestic pigs has remained highly under-researched. Pig keeping in Uganda is historically recent, and evidence on zoonotic pig diseases, including infection with *Trichinella* species, is scarce. A cross-sectional survey on *Trichinella* seroprevalence in pigs was conducted in three districts in Central and Eastern Uganda from April 2013 to January 2015. Serum from a random sample of 1125 pigs from 22 villages in Eastern and Central Uganda was examined to detect immunoglobulin G (IgG) against any *Trichinella* spp. using a commercially available ELISA based on excretory-secretory antigen. ELISA positive samples were confirmed using Western Blot based on somatic antigen of *Trichinella spiralis* as recommended in previous validation studies. Diaphragm pillar muscle samples (at least 5 g each) of 499 pigs from areas with high ELISA positivity were examined using the artificial digestion method. Overall, 78 of all 1125 animals (6.9%, 95% CI: 5.6–8.6%) tested positive for antibodies against *Trichinella* spp. in the ELISA at significantly higher levels in Kamuli district compared to Masaka and Mukono districts. Thirty-one percent of the ELISA positive samples were confirmed IgG positive by the Western Blot leading to an overall seroprevalence of 2.1% (95% CI: 1.4–3.2%). The large proportion of ELISA positive samples that could not be confirmed using Western blot may be the result of cross-reactivity with other gastrointestinal helminth infections or unknown host-specific immune response mechanisms in local pig breeds in Uganda. Attempts to isolate muscle larvae for species determination using the artificial digestion method were unsuccessful. Due to the large number of muscle samples examined we are confident that even if pigs are infected, the larval burden in pork is too low to pose a major risk to consumers of developing trichinellosis. This was the first large systematic field investigation of *Trichinella* infection in domestic pigs in Uganda and its results imply that further studies are needed to identify the *Trichinella* species involved, and to identify potential sources of infection for humans.

## Introduction

Human trichinellosis is acquired through the ingestion of the first-stage larvae of the nematode *Trichinella* in raw or undercooked meat from domestic animals, mainly pigs, and game. Clinically relevant infections can initially cause diarrhoea and abdominal pain; subsequently, migrating larvae and their metabolites may provoke fever, facial oedema and myalgia, or even fatal myocarditis. The severity of the symptoms may depend on the infection dose and the *Trichinella* spp. causing the infection [1].

The nematode *Trichinella* is cosmopolitan and found everywhere except the Antarctica. Eight species and four genotypes have so far been documented, five of them in Africa: *T*. *britovi* in North and West Africa, *Trichinella* T8 in Namibia and South Africa, *T*. *nelsoni* in Eastern and South Africa, *T*. *zimbabwensis* in Southern and Eastern Africa and *T*. *spiralis* in Egypt [2–5]. While a wide range of host species can be infected (mammals, birds and reptiles), only humans show clinical signs in case of infection [1]. While human infection is strictly related to the consumption of raw or undercooked infected meat [3], the major risk factors associated with infection in pigs are the ingestion of infectious first-stage larvae by scavenging, or feeding on scraps from pig slaughter or game hunting [6].

Most previous research on trichinellosis in Africa focused on isolating *Trichinella* from wildlife [7–13], and it was long believed that in Africa, *Trichinella* infection essentially affects wild carnivores [14] and that pigs are not infected with *Trichinella* in East Africa. Outbreaks of human trichinellosis in Africa date back several decades and were documented in Egypt [15], Northwest Ethiopia [16,17], Kenya [18] and Tanzania [19]. Other cases have been diagnosed in France in travellers returning from Senegal [20], a patient in Japan after travelling to Kenya [21], and travellers returning to the United States of America from Africa [22]. These cases of human trichinellosis have been mostly associated with the consumption of game meat, especially bush pig (*Potamochoerus* sp.) and warthog (*Phacochoerus* sp.). The prevalence in wildlife by regions and countries in Africa has been synthesized in recent reviews [5,8] but the real prevalence of human trichinellosis in Africa in general, and Uganda in particular, remains unknown.

Only two studies on *Trichinella*-specific antibodies in domestic pigs in sub-Saharan Africa have been published, both from Nigeria, and reporting seroprevalences of 40% (48/120) [23] and 11% (49/450) [24]. Attempts to directly detect muscle larvae from potentially naturally infected domestic pigs have been made in Northern Tanzania [25] and Ghana [26], but unsuccessfully. However, in Egypt, where *T*. *spiralis* had previously been documented, muscle larvae could be isolated by means of artificial digestion [15,27].

In sub-Saharan Africa, human population is growing rapidly and urbanization is increasing while most of the food is produced in the rural areas by smallholder farmers. Pig keeping has become a popular income-generating activity across Eastern Africa. However, pigs are not a traditional livestock species in the region and, in Uganda, evidence on zoonotic pig diseases has been scarce and limited to pigs as a reservoir for *Trypanosoma* species as well as the natural intermediate host for *Taenia solium* [28]. The presence of porcine *Trichinella* infection in Uganda was reported to the World Organisation for Animal Health (OIE) in 2008 but without any further provision of details [29].

The objectives of the present survey were to investigate if domestic pigs in Uganda are exposed to *Trichinella* by means of indirect methods in order to estimate if consumers are at risk of contracting trichinellosis through pork consumption.

## Materials and Methods

### Ethics statement

The research involved obtaining information from pig farmers and sampling of live animals as well as meat sampling from butcheries. Approval was obtained from the Research and Ethics Committee at the College of Veterinary Medicine, Animal Resources and Biosecurity, Makerere University, Kampala (Ref.: VAB/REC/13/103) and from the Uganda National Council for Science and Technology (Ref.: A 525). Informed consent was obtained from all individual participants included in the study.

### Study area

A cross-sectional survey was conducted in three districts in Central and Eastern Uganda from April 2013 to January 2015. Uganda is located between 4°N and 1°S of the equator in East Africa at the northern shores of Lake Victoria. Due to its proximity to the equator, the climate is tropical savannah in most parts of the country with abundant vegetation and rainfall. Despite its small size, the country’s fauna is extremely diverse [30] and located in more than 60 protected areas, including ten national parks, and more than 500 forest reserves.

### Site selection

**Fig 1:**
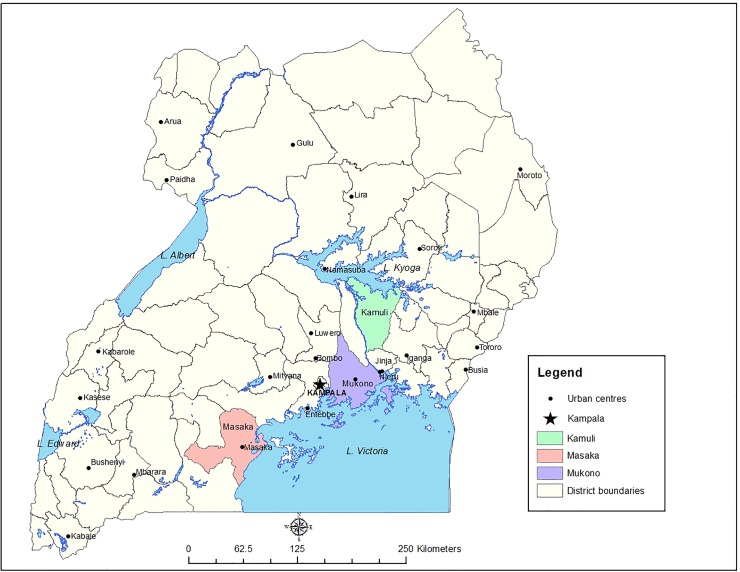
Twenty-two villages were selected for a multi-pathogen survey in pigs in three districts of Central and Eastern Uganda, April to July 2013. (Pamela Ochungo/ ILRI)

As part of a research for development project led by the International Livestock Research Institute (ILRI) and partners, Kamuli, Mukono and Masaka districts were selected with a range of stakeholders. Details of the process and criteria are described elsewhere [31,32]. Based on the 2008 livestock census [33], four to six sub-counties with a high pig population were purposively selected, and villages were further categorized into value chain domains by means of a checklist and consultation with local partners. Value chain domains describe the spatial dimensions of production and consumption of pigs: rural production for rural consumption (rural-rural, RR), rural production for urban consumption (rural-urban, RU) and urban production for urban consumption (urban-urban, UU) [31]. In each district, two sub-counties were purposively selected, and within each sub-county, two to three villages were randomly selected. From a total of 35 villages [31], 22 villages were purposively selected for the present prevalence study (Fig 1), based on available financial and logistic resources.

### Sample size calculation

The original sample size was calculated to estimate district-level prevalence and considering an infinite population using n = [Z^2^P(1–P)]/d^2^; where n is the required sample size; Z is the multiplier from a standard normal distribution (1.96) at a probability level of 0.05; P is the estimated prevalence which is most conservatively estimated to be 50% considering there is no reference data from pigs in the area under study; (1–P) is the probability of having no disease and d is the desired precision level (5%). Therefore, a sample size of 384 pigs per district was required for the study. To increase precision, a sample size of 400 pigs in each district was planned.

### Selection of the individual animals

In each of the 22 selected villages, a list of all households keeping pigs was generated in collaboration with the local government. From that list, households were randomly selected and invited to participate. Per household, one animal was selected for blood collection if it was aged three months or older, not weak or emaciated, not pregnant, and did not have a litter less than two months old.

Pigs were restrained using a snare and bled from the cranial *vena cava* using BD Vacutainer® needles and BD Vacutainer® plain tubes. The blood samples were kept standing in an ice box at 4°C to avoid haemolysis. At the field laboratory, blood was centrifuged and serum harvested into barcoded vials that were stored at -20°C until processed at Makerere University in Kampala, Uganda. Prior to shipment to Friedrich-Loeffler-Institute, Greifswald, Germany, the samples were heat-inactivated at 56°C for 20 minutes.

### Enzyme-linked immunosorbent assay (ELISA)

A commercially available ELISA based on excretory-secretory (E/S) antigen was used to detect immunoglobulin G (IgG) against any *Trichinella* spp. in pig sera (PrioCHECK® Trichinella Ab, Prionics AG, Wagisstrasse 27A, CH-8952 Schlieren). According to the manufacturer, the PrioCHECK® Trichinella Ab ELISA showed a sensitivity and specificity of 100% in an in-house validation study [34].

The assay was performed according to the manufacturer’s instructions in a four-step protocol: serum was diluted 1:50 and incubated on plates coated with E/S antigen of *Trichinella spiralis*; a peroxidase-labelled anti-pig IgG was used as a secondary antibody bound to the antigen; a chromogen (TMB) substrate was added and the optical density of the sample was measured by reading the test plate at 450 nm wavelength (Sunrise™ Tecan, powered by Magellan™ software). The results were calculated in percent positivity (PP) in reference to the positive control sample. Results at or above the manufacturer’s cut-off of 15% PP were considered ELISA positive.

### Confirmatory testing

It is recommended that ELISA positive samples are tested by Western Blot for confirmation of the presence of IgG antibodies against *Trichinella* spp. [35,36]. While the ELISA was based on E/S antigen, the Western Blot used for confirmatory testing in this study was based on somatic antigen from crude worm extract (CWE) of *Trichinella spiralis* first-stage larvae [37]. In addition to all relevant protein fractions of the E/S antigen (43–45 and 66–67 kDa), it includes fractions 47, 61, and 102 kDa which are *Trichinella*-specific [36]. In validation studies evaluating CWE- against E/S-Western Blot and using artificial digestion as ‘gold standard’, the CWE-Western Blot showed a sensitivity ranging from 95.8–91.1% and a specificity ranging from 99.5–100% [35,36]. In this study, it was performed on all ELISA positive samples, and a randomly selected subset of ELISA negative samples (n = 16) for internal quality control. Sera that showed an antibody reaction to any of the five immunogenic protein bands mentioned above were considered Western Blot positive.

### Artificial digestion

In an attempt to isolate *Trichinella* muscle larvae for species determination, 499 pig diaphragm samples from four clusters with a high antibody prevalence based on the ELISA test were collected from October 2014 to January 2015. Diaphragm muscle is the major predilection site for *Trichinella* spp. in domestic swine [38,39]. Artificial digestion according to OIE instructions [40] was carried out at the Central Diagnostic Laboratory at the College of Veterinary Medicine, Animal Resources and Biosecurity, Makerere University in Kampala, Uganda. A positive control of *Trichinella spiralis* was provided by the National Reference Laboratory for *Trichinella* hosted by the Federal Institute for Risk Assessment in Berlin, Germany.

Muscle samples of at least 5 g per animal were tested to increase sensitivity of the artificial digestion method [37,39,41]; 102 samples weighed 5 g; 396 samples 10 g and one sample 20 g. Up to 20 samples with a total weight of 100 g were examined in a pool by artificial digestion method [40].

### Household survey data

Using a structured questionnaire, information on self-reported husbandry and consumption practices was collected at each household from where a pig was sampled.

### Data management

ELISA results were exported to Microsoft Excel, Version 2010, from the ELISA reader using Magellan™ software. Laboratory data from Western Blot and artificial digestion were entered into Microsoft Excel, Version 2010. Household data were entered using Census and Survey Processing System, Version 4.1. (U.S. Census Bureau), and subsequently exported to MySQL for data validation. Datasets were merged for cleaning and descriptive analysis in STATA 13.1 (StataCorp).

## Results

### Antibody prevalence

**Fig 2:**
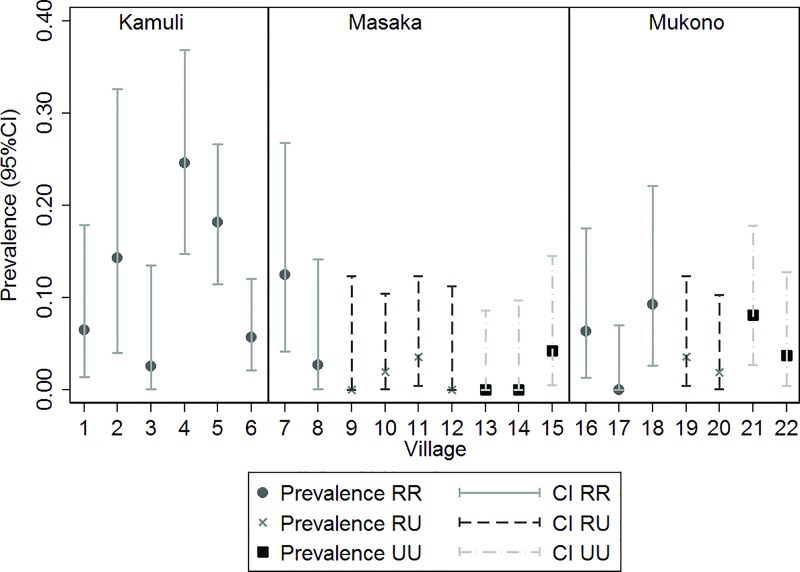
Prevalence estimates and confidence levels for anti-*Trichinella* IgG (PrioCHECK Trichinella Ab) in three districts in Central and Eastern Uganda, sampled between April and July 2013.

Overall, 78 of 1125 animals (6.9%, 95% CI: 5.6–8.6%) tested positive for antibodies against *Trichinella* spp. in the ELISA (Table 1), with significant differences across districts (p < 0.001), sub-counties (p < 0.05) and value chain types (p = 0.05) (Fig 2). Most ELISA positive samples were found in Kamuli district, which is dominated by an RR value chain type, compared to Mukono and Masaka districts.

**Table 1:** Prevalence estimates for anti-*Trichinella* IgG (PrioCHECK^®^ Trichinella Ab) in three districts in Central and Eastern Uganda, sampled between April and July 2013.

District	Village	Value chain type^a^	Sample size (n)	ELISA+	Prevalence estimate^b^ (confidence limits)
Kamuli	(1) Baluboinewa	RR	46	3	6.5% (2.1–18.6%)
** **	(2) Bukyonza B	RR	28	4	14.3% (5.4–32.9%)
** **	(3) Kantu zone	RR	110	20	18.2% (12.0–26.6%)
** **	(4) Ntansi	RR	105	6	5.7% (2.6–12.2%)
** **	(5) Butabala	RR	39	1	2.6% (0.4–16.5%)
** **	(6) Isingo A	RR	65	16	24.6% (15.6–36.6%)
**Total**			**393**	**50**	**12.7% (9.8–16.4%)**
Masaka	(7) Kyamuyimbwa-Kikalala	RU	31	0	0%
** **	(8) Butego	RU	28	0	0%
** **	(9) Kijjabwemi	UU	41	0	0%
** **	(10) Kyabakuza B	UU	36	0	0%
** **	(11) Kisoso	RU	56	2	3.6% (0.9–13.4%)
** **	(12) Ssenya	RR	37	1	2.7% (0.4–17.3%)
** **	(13) Kanoni-Bukunda	RU	51	1	2.0% (0.3–12.9%)
** **	(14) Lukindu	RR	40	5	12.5% (5.2–27.0%)
** **	(15) Ssenyange A	UU	47	2	4.3% (1.1–15.7%)
**Total**			**367**	**11**	**3.0% (1.7–5.3%)**
Mukono	(16) Dundu	RU	56	2	3.6% (0.9–13.4%)
** **	(17) Kyoga	RU	52	1	1.9% (2.7–12.7%)
** **	(18) Joggo	RR	62	5	8.1% (3.4–18.1%)
** **	(19) Kitete	RR	54	2	3.7% (0.9–13.8%)
** **	(20) Bugoye-Kabira	RR	47	3	6.4% (2.1–18.2%)
** **	(21) Kazo-Kalagala	RR	51	0	0%
** **	(22) Nsanja-Gonve	RR	43	4	9.3% (3.5–22.5%)
**Total**			**365**	**17**	**4.7% (2.9–7.4%)**
**Grand Total**			**1125**	**78**	**6.9% (5.6–8.6%)**

^a^Spatial dimensions of production and consumption of pigs [31]: rural production for rural consumption (RR); rural production for urban consumption (RU); urban production for urban consumption (UU)

^b^Calculated at p = 0.05 and CI = 0.95

**Fig 3:**
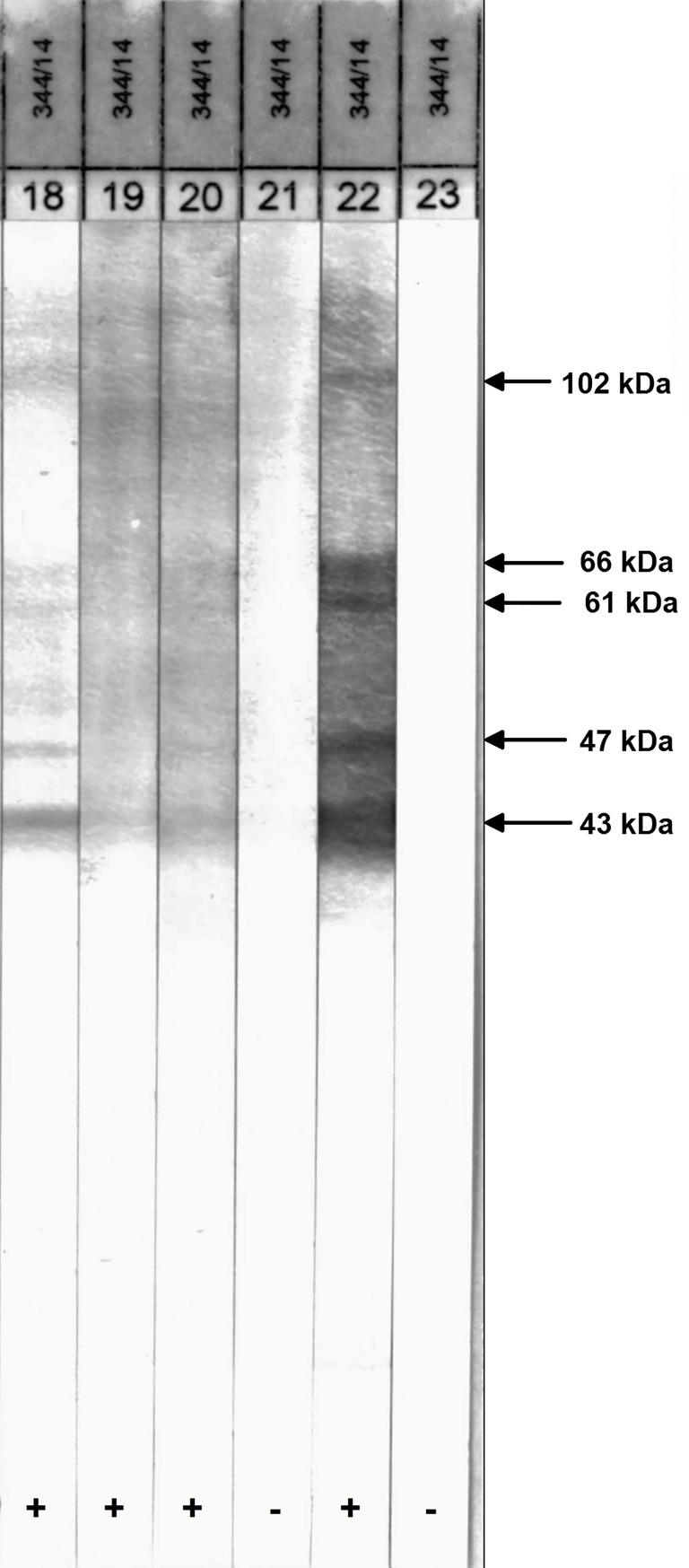
Four of the 78 ELISA positive samples tested by Western Blot based on somatic antigen (strip 18–21). Strip 22 represents the positive control and strip 23 the negative control.

**Fig 4:**
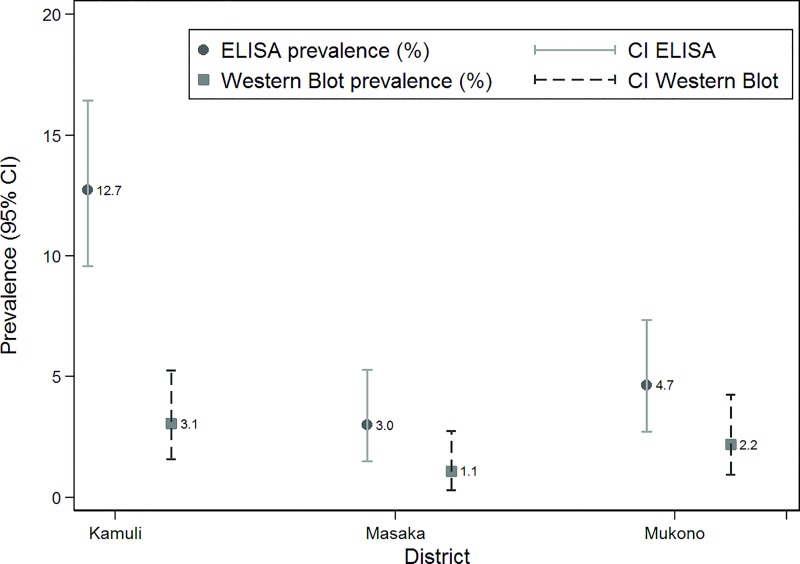
Proportion of ELISA positive samples confirmed by Western Blot based on somatic antigen of *Trichinella spiralis* in three districts in Central and Eastern Uganda, sampled between April and July 2013.

Twenty-four (30.8%) of the 78 ELISA positive samples were confirmed IgG positive by the Western Blot leading to an overall seroprevalence of 2.1% (95% CI: 1.4–3.2%) (Fig 3 and Table 2). There were no significant differences in the Western Blot prevalence between districts (Fig 4). However, the proportion of confirmed samples showed a reverse order with most ELISA positive samples confirmed in Mukono district (47%), followed by Masaka (36%) and Kamuli (24%) districts. All ELISA negative samples used for internal quality control tested negative in the Western Blot.

**Table 2:** Western Blot (WB) confirmation of ELISA positive samples (anti-*Trichinella* IgG), collected between April and July 2013 in 22 villages from three districts in Central and Eastern Uganda.

District	Sample size (n)	ELISA+	WB-	WB+	Total confirmed (%)	WB prevalence estimate^a^ (confidence limits)
Kamuli	393	50	38	12	24.0	3.1% (1.7–5.3%)
Masaka	367	11	7	4	36.4	1.1% (0.4–2.9%)
Mukono	365	17	9	8	47.1	2.2% (1.1–4.3%)
Total	1125	78	54	24	30.8	2.1% (1.4–3.2%)

WB: Western Blot

^a^Calculated at p = 0.05 and CI = 0.95

### Artificial digestion

A total of 499 diaphragm samples were collected from 32 butcheries, representing all butcheries operating in the four sub-counties with the highest ELISA prevalence at the time of sampling. *Trichinella* muscle larvae were not detected in any of the samples.

### Descriptive analysis

Two-thirds of the households participating in the survey were male-headed (67.0%) with a more equal ratio (60:40) in Masaka district. The majority of the households in the study were members of the Christian faith (94.0%) and four pig keeping households (0.4%) in Masaka and Mukono districts were headed by Muslims. In all districts, the most commonly found herd size was two to five pigs (39.4% of households); households keeping only one pig were more common in rural Kamuli (28.5%), while in Masaka and Mukono districts, keeping six to ten pigs was more frequent. The most commonly reared breeds (58.3%) were crosses between the so-called local (black) and exotic (i.e. Large White, Camborough) breeds.

Most of the animals (76.7%) were raised in rural settings. According to the ILRI value chain classification, 30.5% of the pigs kept in rural areas were destined for sale at urban markets (RU) while the rest were intended to be marketed locally (RR). More than half of the households kept their pigs tethered or confined (53.5% and 52.7%, respectively), and only a small fraction left their pigs roam free (2.1%). Tethering was the preferred method of restricting the movement of pigs in Kamuli and Mukono districts, while in Masaka almost three-quarters of pig farmers kept their pigs confined. Overall, all pig farming households fed mixed pig rations, utilizing crop residues (98.3%) and commercial feeds (70.9%), while 35.0% practised swill feeding (e.g. kitchen or restaurant leftovers and wasted bread). More than half of the pig farming households (52.9%) performed routine pest/rodent control.

Direct and indirect contact with wild animals was proxied by reports of wildlife sightings in the village (34.9% of pig farmers). However, only 4.1% of pig farmers reported that they consumed game meat. Those who did ate meat from antelopes such as Sitatunga (*Tragelaphus spekii*) or Ugandan Kob (*Kobus kob thomasi*), but also rodents such as cane rats (*Thryonomys* spp., locally referred to as “edible rats”), or *Canidae* including stray dogs (*Canis familiaris*) and civet cats (*Viverridae*) as well as wild pigs (*Potamochoerus* spp.), buffaloes (*Syncerus caffer*), hares (*Leporidae*) and guinea fowls (*Numididae*).

Slaughtering pigs at home was more frequent in Masaka and Mukono districts. Overall, only 12.6% of pig farmers slaughtered at home at least once a year; the majority (71.2%) never slaughtered their own pigs (Table 3). Of those farmers who slaughtered at home, more than three-quarters reported that the meat had never been inspected. At slaughter, viscera and intestinal contents were usually buried, thrown into the bush or given to dogs. Most of the pig farmers in the study area were also pork consumers and ate pig meat at least once a month (57.8%), while 21.7% never consumed pork and 15.4% ate it only occasionally. The most frequent mode of processing the meat was frying (62.9%) or boiling (37.7%) (Table 3).

**Table 3:** Pig slaughter, processing and consumption practices in Central and Eastern Uganda (2013).

Variable	Totals n (%)
*Frequency of home slaughter*	
≥ Once a month	26 (2.74%)
≥ Once a year	71 (7.49%)
≤ Once a year	22 (2.32%)
Never	675 (71.20%)
Cannot remember/don’t know	9 (0.95%)
Missing	145 (15.30%)
Total	948 (100.00%)
*Frequency of meat inspection at home slaughter (if practiced)*	
Always	7 (5.88%)
Sometimes	9 (7.56%)
Cannot remember/don’t know	2 (1.68%)
Never	93 (76.15%)
Missing	8 (6.72%)
Total	119 (100.00%)
*Disposal of viscera & intestinal contents*	
Throw away outside compound	17 (1.79%)
Throw away inside compound	2 (0.21%)
Manure	0
Feed the live pigs	0
Other: buried	28 (2.95%)
Other: given to dogs	12 (1.27%)
Other: given to people	2 (0.21%)
Other: not specified	81 (8.54%)
Missing	806 (85.02%)
Total	948 (100.00%)
*Frequency of pork consumption*	
≥ Once a month	548 (57.81%)
≥ Once a year	146 (15.40%)
≤ Once a year	32 (3.38%)
Never	206 (21.73%)
Missing	16 (1.69%)
Total	948 (100.00%)
*Pork preparation*	
Boiling	357/948 (37.66%)
Frying	596/948 (62.87%)
Barbeque	95/948 (10.02%)
Other	7/948 (0.74%)
Missing	213/948 (22.47%)
*Consumption of game meat*	
	39/948 (4.11%)

## Discussion

The present survey is one of the few in sub-Saharan Africa and the first in Uganda to investigate the occurrence of infection with *Trichinella* spp. in domestic pigs. To the authors’ knowledge, it is also the first investigation in Africa confirming ELISA positive results by Western Blot that resulted in an overall seroprevalence of 2.1%.

Two farm surveys in Nigeria found a prevalence of *Trichinella* IgG of 10.9% [24] and 40% [23]. Both surveys used the same commercial ELISA based on E/S antigens as in the present survey but reported higher prevalence rates. In previous studies, this ELISA detected antibodies in samples with larval densities of 0.025 larvae per gram (LPG) [42]. The authors of those validation studies were also able to detect antibodies in pigs infected with different *Trichinella* species, like *T*. *spiralis*, *T*. *britovi* and *T*. *pseudospiralis*, but showed no cross-reactivity with other pig pathogens such as *Trichuris suis*, *Oesophagostomum*, *Strongyloides*, *Hyostrongylus*, *Oesophagostomum*/*Hyostrongylus* and *Salmonella typhimurium* [34]. *T*. *nelsoni*, a *Trichinella* species previously reported in game animals in Eastern Africa, has not been included in those validation studies.

ELISAs of the preceding generation were based on somatic antigen of *Trichinella spiralis*. Since then, the specificity of the ELISA has been improved by utilizing E/S antigens obtained from metabolites of *Trichinella* muscle larvae incubated *in vitro* [37]. While the manufacturer of the commercial kit used in this study reports a sensitivity and specificity of 100% [34], a comparable in-house ELISA for the detection of *Trichinella*-specific antibodies had a test sensitivity and specificity of 96.8% and 97.9%, respectively [36]. As shown in other studies, unspecific cross-reactions may not be fully excluded; therefore, a Western Blot was used for confirmatory testing. Reportedly, the combined use of both serological methods shows a sensitivity that is 31.4 times higher than that of the digestion assay [43], especially in naturally infected wild pigs with a lower larval burden. Western Blot based on somatic antigen allows discriminating ELISA positive samples further because it includes all relevant protein fractions of the E/S antigen (43–45 and 66–67 kDa) and fractions 47, 61 and 102 kDa which are *Trichinella*-specific [35,36]. The obvious discrepancy between ELISA positives and Western Blot positives may be the result of cross-reactivity with other gastrointestinal helminth infections as found in this study for co-infection with strongyle species (data not shown). It could also be caused by unknown host-specific immune response mechanisms in local pig breeds from Uganda because the validation studies involved European pig breeds.

We were not able to determine the *Trichinella* species infecting domestic pigs in the investigated study areas of Uganda as artificial digestion of a large number of muscle samples did not result in the isolation of muscle larvae. Infectivity and larval burden in domestic pigs largely depends on the *Trichinella* species. While *T*. *spiralis* has a very high reproductive capacity resulting in 427.4 LPG five weeks p.i., *T*. *nelsoni* and *T*. *britovi* result in a larval burden of 52.2 and 38.0 LPG, respectively [44]. This may explain why potentially low larval burdens of a non-*T*. *spiralis* infestation in the muscle could not be detected by artificial digestion. For future studies, testing larger amounts (up to 50–100g) of muscle tissue may compensate for a possible decrease in sensitivity [40] due to unknown larval burden in predilection sites. In addition, future sampling studies to detect larvae should concentrate in areas where seroprevalence was highest by Western Blot. This was not applicable in this study due to logistics and timing of sampling and testing.

While the Nigerian survey by Momoh et al. [23] reported potential risk factors, we deliberately waived statistical risk factor analysis in our study. We performed Western Blot as a confirmatory test which reduced the number of positive samples by 69% and left us with too few positives to conduct sound univariate analysis. Confirmation by Western Blot has not been done in previous studies in domestic pigs in Africa where ELISA positivity was used as the outcome variable and the number of observations was much higher. In the present study, the outcome variable would ideally be the result of the Western Blot. In this study, 78 samples tested ELISA positive and 24 were confirmed as Western Blot positive; 1047 samples tested ELISA negative but an unknown number could be Western Blot positive. Since we had only sufficient funding to carry out Western Blot on a subset of 16 ELISA negative samples, and although all were negative in Western Blot, it is not possible to definitively identify which (if any) of the untested 1031 ELISA negative samples would have tested positive or negative in Western Blot. Thus, conducting a risk factor analysis on the small subset of ELISA positive samples was not considered meaningful as the relation between ELISA positive, Western Blot positive, ELISA negative and Western Blot negative samples was not known, and risk factor analysis would then only apply to a small subset of pigs of unknown epidemiological status.

From the information gathered in this survey, potential reservoirs for *Trichinella* sp. such as rodents and stray dogs should be investigated in the future. They may act as potential links between the sylvatic and domestic life cycle as shown by researchers in Egypt who isolated muscle larvae from dogs and rodents in urban settings and in the proximity of abattoirs [45,46].

Information gathered on slaughter and consumption practices are indicative but not conclusive. However, previous reports in the same geographical area suggest that pig farmers do not consume raw pork but thoroughly heat meat products [47]. Results on slaughter practices, for instance, disposal of remains, or the consumption of game meat, are likely to be underreported by the participants of the present survey. The frequency of wildlife sightings, too, may be underreported as wildlife is very common in some areas and often only subconsciously noticed as it is such an integral part of daily life.

Previous reviews suggest that *Trichinella* infection in pigs and people plays a negligible role in Africa because the people of sub-Saharan Africa consist mainly of Bantu origin who do not consume meat [6,48]. Personal observation of an increasing number of roasted meat outlets (both pork and beef) and Food and Agriculture Organization of the United Nations statistics on per capita consumption of meat [49] strongly disagree with this argument. In Uganda, pig numbers increased more than tenfold from 200,000 to more than 3 million since the 1980s [33], while per capita consumption is highest in Eastern Africa at 3.4 kg [49] at increasing trends. In general, the history of the domestic pig in Africa is highly controversial and has been heavily neglected in research [50]. Uganda in particular is a very diverse country with Muslims and Christians intermarrying and a substantial inflow of migrants, especially from China and India [51], who traditionally consume a lot of pork and may contribute to an increased demand in the near future.

In conclusion, we were able to demonstrate that domestic pigs in Kamuli, Masaka and Mukono districts in Uganda have been infected with *Trichinella* spp. This was the first large systematic investigation of *Trichinella* infection in domestic pigs in Uganda, potentially identifying high-risk areas through the confirmation of ELISA positive samples using Western Blot. We were not able to identify the species; however, this was not the objective of the study. Due to the large number of diaphragm muscle samples examined at higher sample weight (at least 5 g), we are confident that even if pigs are infected, the larval burden in pork is too low to pose a major risk to consumers of developing trichinellosis. Further studies are needed to identify the *Trichinella* species infecting domestic pigs and to identify potential sources of infection and modes of transmission.

## References

[1] Gottstein B, Pozio E, Nöckler K. Epidemiology, diagnosis, treatment, and control of trichinellosis. Clin Microbiol Rev. 2009;22: 127–145. 10.1128/CMR.00026-08 19136437 PMC2620635

[2] Pozio E, Pagani P, Marucci G, Zarlenga DS, Hoberg EP, De Meneghi D, et al Trichinella britovi etiological agent of sylvatic trichinellosis in the Republic of Guinea (West Africa) and a re-evaluation of geographical distribution for encapsulated species in Africa. Int J Parasitol. 2005;35: 955–960. 10.1016/j.ijpara.2005.03.013 15964575

[3] Pozio E. World distribution of *Trichinella* spp. infections in animals and humans. Vet Parasitol. 2007;149: 3–21. 10.1016/j.vetpar.2007.07.002 17689195

[4] Murrell KD, Pozio E. Worldwide occurrence and impact of human trichinellosis, 1986–2009. Emerg Infect Dis. 2011;17: 2194–2202. 10.3201/eid1712.110896 22172230 PMC3311199

[5] Mukaratirwa S, La Grange L, Pfukenyi DM. *Trichinella* infections in animals and humans in sub-Saharan Africa: A review. Acta Trop. Elsevier B.V.; 2013;125: 82–89. 10.1016/j.actatropica.2012.09.005 23041114

[6] Dupouy-Camet J, Murrell K. FAO/WHO/OIE Guidelines for the surveillance, management, prevention and control of trichinellosis. Paris, France; 2007.

[7] Pozio E, De Meneghi D, Roelke-Parker ME, La Rosa G. *Trichinella nelsoni* in carnivores from the Serengeti ecosystem, Tanzania. J Parasitol. 1997;83: 1195–1198. 10.2307/3284388 9406805

[8] Pozio E, Foggin CM, Gelanew T, Marucci G, Hailu A, Rossi P, et al *Trichinella zimbabwensis* in wild reptiles of Zimbabwe and Mozambique and farmed reptiles of Ethiopia. Vet Parasitol. 2007;143: 305–310. 10.1016/j.vetpar.2006.08.029 16982152

[9] Marucci G, La Grange LJ, La Rosa G, Pozio E. *Trichinella nelsoni* and *Trichinella* T8 mixed infection in a lion (Panthera leo) of the Kruger National Park (South Africa). Vet Parasitol. 2009;159: 225–8. 10.1016/j.vetpar.2008.10.041 19041185

[10] La Grange LJ, Marucci G, Pozio E. *Trichinella zimbabwensis* in wild Nile crocodiles (*Crocodylus niloticus*) of South Africa. Vet Parasitol. 2009;161: 88–91. 10.1016/j.vetpar.2008.12.015 19167165

[11] La Grange LJ, Marucci G, Pozio E. *Trichinella zimbabwensis* in a naturally infected mammal. J Helminthol. 2010;84: 35–38. 10.1017/S0022149X09990241 19580688

[12] La Grange LJ, Govender D, Mukaratirwa S. The occurrence of *Trichinella zimbabwensis* in naturally infected wild crocodiles (*Crocodylus niloticus*) from the Kruger National Park, South Africa. J Helminthol. 2013;87: 91–96. 10.1017/S0022149X12000089 22335961

[13] La Grange LJ, Reininghaus B, Mukaratirwa S. First report of a mixed infection of *Trichinella nelsoni* and *Trichinella* T8 in a leopard (Panthera pardus) from the Greater Kruger National Park, South Africa. Onderstepoort J Vet Res. 2014;81 Available: http://www.ncbi.nlm.nih.gov/pubmed/25686214 10.4102/ojvr.v81i1.836 25686214

[14] Young E, Whyte IJ. Trichinosis (*Trichinella spiralis* infestations) in wild animals of the Kruger National Park. J S Afr Vet Assoc. 1975;46: 233–234. Available: http://www.ncbi.nlm.nih.gov/pubmed/1240968 1240968

[15] Sayed A, Hussein A, Arafa M, Abdo B. Epidemiological study on trichinellosis in pigs and man in Upper Egypt. Assiut Vet Med J. 2010;56: 280–287.

[16] Kefenie H, Bero G. Trichinosis from wild boar meat in Gojjam, north-west Ethiopia. Trop Geogr Med. 1992;44: 278–280. Available: http://www.ncbi.nlm.nih.gov/pubmed/1455537 1455537

[17] Kefenie H, Wolde H, Abuherpo M. Trichinosis from wild boar meat in Arsi, central Ethiopia. Ethiop Med J. 1988;26: 97–100. Available: http://www.ncbi.nlm.nih.gov/pubmed/3360005 3360005

[18] Kaminsky RG, Zimmerman RR. *Trichinella spiralis*: incidental finding. East Afr Med J. 1977;54: 643–646. Available: http://www.ncbi.nlm.nih.gov/pubmed/614130 614130

[19] Bura MW, Willett WC. An outbreak of trichinosis in Tanzania. East Afr Med J. 1977;54: 185–193. Available: http://www.ncbi.nlm.nih.gov/pubmed/561687 561687

[20] Dupouy-Camet J, Lecam S, Talabani H, Ancelle T. Trichinellosis acquired in Senegal from warthog ham, March 2009. Bull Eur sur les Mal Transm. 2009;14 Available: http://www.ncbi.nlm.nih.gov/pubmed/19480811 10.2807/ese.14.21.19220-en 19480811

[21] Nakamura T, Miura T, Nakaoka T, Nagano I, Takahashi Y, Iwamoto A. [A case of trichinellosis with spontaneous remission]. Kansenshōgaku zasshi J Japanese Assoc Infect Dis. 2003;77: 839–843. Available: http://www.ncbi.nlm.nih.gov/pubmed/14608917 10.11150/kansenshogakuzasshi1970.77.839 14608917

[22] McAuley JB, Michelson MK, Schantz PM. *Trichinella* infection in travelers. J Infect Dis. 1991;164: 1013–6. Available: http://www.ncbi.nlm.nih.gov/pubmed/1940453 1940453 10.1093/infdis/164.5.1013

[23] Momoh HA, Bello M, Inabo H, Wada Y, Adole EB, Madaiki BD, et al Prevalence and some risk factors associated with trichinellosis in backyard pig farms in Zaria, Nigeria. Trop Anim Health Prod. 2013;45: 1149–1152. 10.1007/s11250-012-0338-3 23264057

[24] Adediran O, Uwalaka E, Abiola J. Seroprevalence of *Trichinella* antibodies in pigs reared under different husbandry systems in Southwestern Nigeria. Alexandria J Vet Sci. 2015;47: 183 10.5455/ajvs.191632

[25] Ngowi HA, Kassuku AA, Maeda GEM, Boa ME, Willingham AL. A slaughter slab survey for extra-intestinal porcine helminth infections in northern Tanzania. Trop Anim Health Prod. 2004;36: 335–340. 10.1023/B:TROP.0000026663.07862.2a 15241967

[26] Permin A, Yelifari L, Bloch P, Steenhard N, Hansen N., Nansen P. Parasites in cross-bred pigs in the Upper East Region of Ghana. Vet Parasitol. 1999;87: 63–71. 10.1016/S0304-4017(99)00159-4 10628701

[27] Morsy TA, Ibrahim BB, Haridy FM, Rifaat MM. *Trichinella* encysted larvae in slaughtered pigs in Cairo (1995–1999). J Egypt Soc Parasitol. 2000;30: 753–760. Available: http://www.ncbi.nlm.nih.gov/pubmed/11198373 11198373

[28] Alonso S, Lindahl J, Roesel K, Traore SG, Yobouet BA, Ndour APN, et al Where literature is scarce: observations and lessons learnt from four systematic reviews of zoonoses in African countries. Anim Heal Res Rev. 2016;17: 28–38. 10.1017/S1466252316000104 27427191

[29] OIE. Detailed country(ies) disease incidence. In: World Animal Health Information Database (WAHIS Interface) [Internet]. 2016 [cited 1 Jan 2016]. Available: http://www.oie.int/wahis_2/public/wahid.php/Diseaseinformation/statusdetail#

[30] WCS. Biodiversity Conservation–the importance of Uganda In: Wildlife Conservation Society [Internet]. 2016 [cited 1 Jan 2016]. Available: http://uganda.wcs.org/Wildlife/Biodiversity.aspx

[31] Ouma E, Dione M, Lule P, Roesel K, Pezo D. Characterization of smallholder pig production systems in Uganda: constraints and opportunities for engaging with market systems. Livest Res Rural Dev. 2014;26: Article #56, retrieved from http://www.lrrd.org/lr. Available: http://www.lrrd.org/lrrd26/3/ouma26056.htm

[32] Dione MM, Ouma EA, Roesel K, Kungu J, Lule P, Pezo D. Participatory assessment of animal health and husbandry practices in smallholder pig production systems in three high poverty districts in Uganda. Prev Vet Med. Elsevier B.V.; 2014;117: 565–76. 10.1016/j.prevetmed.2014.10.012 25458705

[33] MAAIF/UBOS. The National Livestock Census Report 2008. Entebbe/Kampala, Uganda; 2009.

[34] Buholzer P, Price PC, Zwald D, Haupt-Gerber T, Bonilla W, Raeber AJ. PrioCHECK® Trichinella Ab, a newly highly sensitive and specific ELISA for the detection of antibodies against Trichinella spp. in serum and meat juice of pigs. In: Richey M, editor. 111th meeting of the United States Animal Health Association. Reno, Nevada: Rapid Solutions Group, Kansas City, Missouri; 2008. pp. 116–130. Available: https://www.google.com/?gws_rd=ssl#q=USAHA+trichinella+buholzer

[35] Frey CF, Schuppers ME, Nöckler K, Marinculić A, Pozio E, Kihm U, et al Validation of a Western Blot for the detection of anti-*Trichinella* spp. antibodies in domestic pigs. Parasitol Res. 2009;104: 1269–1277. 10.1007/s00436-008-1321-9 19130084

[36] Nöckler K, Reckinger S, Broglia A, Mayer-Scholl A, Bahn P. Evaluation of a Western Blot and ELISA for the detection of anti-*Trichinella*-IgG in pig sera. Vet Parasitol. 2009;163: 341–347. 10.1016/j.vetpar.2009.04.034 19473770

[37] Nöckler K, Pozio E, Voigt W., Heidrich J. Detection of *Trichinella* infection in food animals. Vet Parasitol. 2000;93: 335–350. 10.1016/S0304-4017(00)00350-2 11099846

[38] Kapel CMO, Webster P, Gamble HR. Muscle distribution of sylvatic and domestic *Trichinella* larvae in production animals and wildlife. Vet Parasitol. 2005;132: 101–105. 10.1016/j.vetpar.2005.05.036 15979801

[39] Gamble HR, Bessonov AS, Cuperlovic K, Gajadhar AA, van Knapen F, Noeckler K, et al International Commission on Trichinellosis: recommendations on methods for the control of *Trichinella* in domestic and wild animals intended for human consumption. Vet Parasitol. 2000;93: 393–408. Available: http://www.ncbi.nlm.nih.gov/pubmed/11099850 11099850 10.1016/s0304-4017(00)00354-x

[40] OIE. Trichinellosis (infection with *Trichinella* spp.). OIE Terrestrial Manual. Organisation Mondiale de la Santé Animale/ World Organisation for Animal Health (OIE); 2012 pp. 305–313. Available: http://www.oie.int/international-standard-setting/terrestrial-manual/access-online/

[41] Forbes LB, Gajadhar AA. A validated *Trichinella* digestion assay and an associated sampling and quality assurance system for use in testing pork and horse meat. J Food Prot. 1999;62: 1308–1313. Available: http://www.ncbi.nlm.nih.gov/pubmed/10571321 10571321 10.4315/0362-028x-62.11.1308

[42] Frey CF, Buholzer P, Beck R, Marinculic A, Raeber AJ, Gottstein B, et al Evaluation of a new commercial enzyme-linked immunosorbent assay for the detection of porcine antibodies against *Trichinella* spp. J Vet Diagn Invest. 2009;21: 692–697. 10.1177/104063870902100516 19737767

[43] Gómez-Morales M, Ludovisi A, Amati M, Bandino E, Capelli G, Corrias F, et al Indirect versus direct detection methods of *Trichinella* spp. infection in wild boar (Sus scrofa). Parasit Vectors. 2014;7: 171 10.1186/1756-3305-7-171 24708795 PMC3995759

[44] Kapel CM, Gamble HR. Infectivity, persistence, and antibody response to domestic and sylvatic *Trichinella* spp. in experimentally infected pigs. Int J Parasitol. 2000;30: 215–21. Available: http://www.ncbi.nlm.nih.gov/pubmed/10704604 10704604 10.1016/s0020-7519(99)00202-7

[45] Mikhail EM, Mansour NS, Awadalla HN. Identification of *Trichinella* isolates from naturally infected stray dogs in Egypt. J Parasitol. 1994;80: 151–4. Available: http://www.ncbi.nlm.nih.gov/pubmed/8308650 8308650

[46] Loutfy NF, Awad OM, El-Masry AG, Kandil GM. Study on rodents infestation in Alexandria and prevalence of *Trichinella spiralis* infection among them. J Egypt Soc Parasitol. 1999;29: 897–909. Available: http://www.ncbi.nlm.nih.gov/pubmed/12561929 12561929

[47] Roesel K, Ouma EA, Dione MM, Pezo D, Clausen P-H, Grace D. Smallholder pig producers in Uganda and their pork consumption practices. The 6th All Africa Conference on Animal Agriculture Theme: Africa’s Animal Agriculture: Macro-trends and future opportunities. Nairobi, Kenya; 2014. pp. 166–168. Available: https://cgspace.cgiar.org/handle/10568/51344

[48] Bruschi F. Trichinellosis in developing countries: is it neglected? J Infect Dev Ctries. 2012;6: 216–22. Available: http://www.ncbi.nlm.nih.gov/pubmed/22421602 22421602 10.3855/jidc.2478

[49] FAOSTAT. Food Balance/ Food Supply [Internet]. 2011 [cited 1 Jan 2016]. Available: http://faostat3.fao.org/browse/FB/CL/E

[50] Blench R. A history of pigs in Africa In: Blench R, MacDonald K, editors. Origins and development of African livestock: Archaeology, genetics, linguistics and ethnography. Oxfordshire: Routledge; 2000 pp. 355–367.

[51] IOM. Migration in Uganda: A Rapid Country Profile 2013. [Internet]. Kampala, Uganda; 2015. Available: http://uganda.iom.int/publication/

